# Integrative genomics approaches validate *PpYUC11-like* as candidate gene for the stony hard trait in peach (*P. persica* L. Batsch)

**DOI:** 10.1186/s12870-018-1293-6

**Published:** 2018-05-18

**Authors:** Marco Cirilli, Daniela Giovannini, Angelo Ciacciulli, Remo Chiozzotto, Stefano Gattolin, Laura Rossini, Alessandro Liverani, Daniele Bassi

**Affiliations:** 1 Department of Agricultural and Environmental Sciences (DISAA), University of Milan, Via Celoria 2, Milan, Italy; 2 CREA Research Centre for Olive, Citrus and Tree Fruit, via La Canapona 1bis, Forlì, Italy; 3 Parco Tecnologico Padano, Via Einstein, Loc. C.na Codazza, Lodi, Italy

**Keywords:** Stony hard, Peach, GWAS, Genomics, Texture

## Abstract

**Background:**

Texture is one of the most important fruit quality attributes. In peach, stony hard (SH) is a recessive monogenic trait (*hd/hd*) that confers exceptionally prolonged firm flesh to fully ripe fruit. Previous studies have shown that the SH mutation affects the fruit ability to synthesize appropriate amounts of indol-3-acetic acid (IAA), which orchestrates the ripening processes through the activation of system 2 ethylene pathway. Allelic variation in a TC microsatellite located within the first intron of *PpYUC11-like* (a YUCCA-like auxin-biosynthesis gene) has been recently proposed as the causal mutation of the SH phenotype.

**Results:**

The simple genetic determinism of the SH trait has been clarified through genome-wide association and LD analyses in a diverse set of accessions, restricting the *hd* locus to an interval of about 1.8 Mbp in chromosome 6. The comparison of fruit transcriptome data from non-SH (melting flesh) and SH accessions provided an expression patterns overview of the annotated transcripts within the *hd* locus, confirming the absence of *PpYUC11-like* expression in SH fruits. To explore further possible associations between genomic variants at the *hd* locus and the SH phenotype, re-sequencing data of the SH accession ‘D41–62’ were compared with several SH and non-SH accessions with different genetic backgrounds. A further step of validation was provided through the evaluation of variant-trait association in two bi-parental F_2_ populations issued from the SH accession ‘D41–62’ and a panel of advanced breeding selections, showing perfect co-segregation of the *PpYUC11-like* intron TC_20_ allele and the SH phenotype.

**Conclusions:**

In this study, we provide a multi-level validation of the genetic control of the SH trait through the integration of genome-wide association mapping, transcriptome analysis and whole-genome resequencing data for SH and non-SH accessions, and marker-trait association in a panel of advanced breeding selections and segregating progenies. Collectively, our data confirm with high confidence the role of allelic variation at *PpYUC11-like* locus as the genetic determinant of the SH trait, opening interesting perspectives at both biological and applied research level.

**Electronic supplementary material:**

The online version of this article (10.1186/s12870-018-1293-6) contains supplementary material, which is available to authorized users.

## Background

Texture is an important fruit quality trait, affecting consumers’ degree of liking and marketability. During ripening, fruits undergo progressive modifications of textural characteristics, caused by structural rearrangement of cell wall polymers and change in turgor pressure, finally leading to fruit softening [30]. In climacteric fruits, ethylene orchestrates ripening processes, activating a cascade of gene pathways through a complex interplay with other hormones [21]. The perturbation of auxin metabolism and/or its signal transduction affects various aspects of fruit ripening and texture changes. For example, down-regulation of *DR12* (an Auxin Response Factor) in tomato induces modification of pectin structure [17]. Application of synthetic auxin in kiwifruit delayed fruit softening, via the reduction of ethylene biosynthesis [7]. In apple, auxin seems to interfere with ethylene perception at receptor level, ensuring the proper timing of ethylene biosynthesis activation [34]**.**

The availability of genetic and genomic resources, and the wide phenotypic variability for textural characteristic, make peach (*P. persica* L. Batsch) an interesting model species for the dissection of the genetic determinism of fruit texture and softening behaviour [5, 28]. The vast majority of peach commercial cultivars are characterized by a melting flesh (MF) texture type, featuring an initial slow decrease of firmness followed by a rapid softening (melting phase), concomitant to the climacteric respiration and ethylene burst [22]. Textural changes in peach involve integrated mechanisms of cell-wall modification and loss of intracellular adhesion (mainly regulated by the activity of endopolygalacturonase enzymes), and loss of turgor pressure, finally resulting in fruit melting and firmness reduction [8, 13, 14]. The MF trait is regulated by a major locus (*M/m*) located on chromosome 4 [31], and harbouring two genes belonging to the endo-PG family, under the control of ethylene signalling pathways [10]. Copy number variation at the *M* locus and involving the presence/absence of two endo-PG isoform (*endoPGM* and *endoPGF*), has been recently demonstrated as the genetic basis of the recessive non-melting flesh (NMF) trait [16]. NMF peach are characterized by a slow decrease of firmness during ripening and the maintenance of a rubbery texture in full ripe fruits [33].

Stony-hard (SH) flesh is a monogenic recessive trait firstly reported by Yoshida [41] and regulated by the *hd* locus. SH fruits produce null or very low ethylene amounts during ripening [18], maintaining a very firm, crunchy apple-like flesh even when fully ripe. The inability to produce ethylene is caused by a low expression of the main ripening-related ethylene biosynthesis gene, the ACC synthase isoform *PpACS1* [35]. SH texture is inherited independently of the MF/NMF flesh trait, but it is epistatic to the latter. The *M/m* locus status is phenotypically revealed only by cold temperature exposure (below 10 °C) of SH fruits or by the treatment with ethylene or its precursor 1-aminocyclopropane-1-carboxylate synthase (ACC) [18, 19]. In contrast to MF peach fruit, characterized by a sharp increase of indol-3-acetic acid (IAA) content at pre-ripening stage, SH fruit fail to up-regulate IAA levels [36]. Exogenous application of a synthetic auxin (1-naphtalene acetic acid, NAA) is able to induce *PpACS1* expression in SH fruits, indicating that proper IAA levels are required for activating ethylene biosynthesis [36]. Among transcripts putatively involved in auxin homeostasis, the expression pattern of *PpYUC11-like* (ppa008176) is strongly correlated with IAA levels at late stages of ripening, while being undetectable in SH fruits [29]. This gene encodes a flavin mono-oxygenase protein similar to the Arabidopsis YUCCA11 protein [12]. In MF fruits, *PpYUC11-like* expression and IAA levels are correlated with *PpACS1* activation, ethylene biosynthesis and firmness reduction. Molecular analysis identified allelic variation in repeat number at a TC microsatellite located in the first intron of *PpYUC11-like* gene: the TC_20_ allele is homozygous in SH accessions and heterozygous or absent in MF ones. Interestingly, in the heterozygous ‘Goldhoney3’ accession, the allele carrying the TC_20_ variant is not transcribed, further supporting its role in conferring the SH phenotype [29].

In spite of the physiological and molecular evidences about the role of this allelic variant in conferring the SH phenotype, a more in-depth validation at genomic level and in a broader genetic background is still lacking. In this work, a comprehensive validation of the causal sequence variation conferring the SH trait is reported by the integration of genome-wide association mapping, transcriptome analysis, whole-genome re-sequencing and variant-trait association approaches in a panel of peach accessions, breeding selections and segregating progenies.

## Methods

### Genome-wide association analysis

The panel used for GWAS was established by including a total of 87 accessions, of which 63 MF, 12 NMF and 12 SH (Additional file 1: Table S1), genotyped with the IPSC peach 9 K SNP array [39]. SNPs were filtered by using the selection criteria previously described [27]. Genotyping data were filtered for marker missing rate < 10% and minor allele frequency (MAF) > 5%, finally retaining a total of 6049 SNPs for GWAS. The Peach Genome assembly V2.0 [40] was used as a reference for SNP marker positions. Phenotypes were coded as binary trait, assigning 1 to SH and 0 to non-SH accessions. Population structure was inferred by using ADMIXTURE v1.22 [1] by inputting successive values of K from 2 to 6. The K value (number of a priori cluster) was chosen based on a 10-fold cross-validation procedure with 10 different fixed initial seeds. For association analysis, Mixed Linear Model (MLM) was performed in GAPIT R package [24]. Random effects were included in the mixed models as kinship matrix computed using Identical-By-State (IBS) algorithm, as implemented in EMMAX package [20]. For fixed effects, a Q-matrix using a value of K = 3 was used as covariate for association analysis. The Fixed and random model Circulating Probability Unification (FarmCPU) method was used to further confirm association signals [25]. The performance of all tested GWAS algorithms was evaluated by comparing the observed vs expected *p*-values under null hypothesis, through quantile-quantile (QQ)-plot inspection and considering statistical power against False-Discovery Rate (FDR). A conservative threshold for assessing SNP significance was calculated based on Bonferroni correction for a type I error rate of 0.01. Intra-chromosomal LD patterns were measured and visualized using HAPLOVIEW v4.2 [6].

### RNA-Seq library preparation, sequencing and analysis

RNA-Seq libraries were constructed from samples collected from two different cultivars for each flesh texture phenotype (MF and SH) at Stage III (SIII, pre-climacteric) and Stage IV (SIV, climacteric) of fruit development. Maturity degree was established by monitoring the I_AD_ index (measuring chlorophyll degradation) using DA-meter instrument and firmness decay using a digital Andilog penetrometer equipped with 8-mm plunger (Additional file 2: Figure S1). Each sample was composed by a mixture of small pieces collected from 10fruits. Total mRNA was extracted using the protocol described in Dal Cin et al. [11] and mRNA library prepared and sequenced at IGA Technology Service (Udine, Italy) according to the Illumina Hiseq2000 manufacturer’s instruction. After quality check, raw sequencing data were filtered, trimmed and aligned to the peach reference genome v.2.0 [40] using Bowtie2 v2.1.0. Mapping of RNA-Seq reads was performed using TopHat2 v2.0.21 [38]. The counts of uniquely mapped reads were generated through HTSeq tool [2] using JGI reference peach annotation in *gtf* file format. The standardization of read counts and the analysis of differentially expressed genes were performed in R using *edgeR* and *limma* packages. Gene expression levels were calculated based on reads per kilobase per million of mapped reads (RPKM) and visualized using *heatmap* R package. Differentially expressed genes (DEGs) were identified through non-parametric t-test equivalent using *nparcomp* package, considering the two accessions of each group as biological replicate.

### Quantitative PCR analyses

For qPCR analyses, fruit samples of the four accessions used for RNAseq analyses were collected during SII to SIV stages of development. RNA was extracted from three biological replicates using RNeasy Plant kit (Qiagen) and cDNA synthesized using Superscript III reverse transcriptase (Thermo Fisher Scientific), according to manufacturers’ protocols. Primers for qPCR analysis are listed in Additional file 3: Table S2. Real-time PCR reaction were carried out using Platinum SYBR Green qPCR Supermix (Thermo Fisher Scientific) on a 7300 RT-PCR System (Applied Biosystems). Relative expression levels were calculated using ∆C_t_ method, using Actin (Prupe.6G163400) as reference gene [36].

### Whole-genome re-sequencing of the stony hard accession ‘D41–62’

Whole-genome sequence (WGS) library of the accession ‘D41–62’ was prepared by the Genomics Platform of Parco Tecnologico Padano (Lodi, Italy) with the Illumina Truseq DNA Nano sample prep kit (Illumina, San Diego) following manufacturer’s protocol and sequenced on the Hiseq2000 with paired-end sequencing module using the Truseq SBS kit v3. FASTQ files were obtained with the Illumina CASAVA Pipeline. WGS libraries from the other accessions were retrieved from SRA archive (accession numbers are provided in Additional file 4: Table S3). After cleaning and filtering, reads were trimmed with Trimmomatic v0.32 and mapped using default parameters onto peach reference genome V2.0 using BWA-MEM algorithm, implemented in BWA v.0.6.1 tool [23]. After alignment, an average coverage of 37.4× was estimated by Samtools *mpileup* tool. For variants identification, after duplicate removal and reads indexing with PICARD, a joint-calling approach was performed using Haplotype Caller algorithm in GATK, following Best Practice guidelines. Sequences for predicted peach gene models were retrieved from the Phytozome v12.1 database [15]. Functional annotation of the variants was performed using SNPEffect v2.0 [32].

### Plant material and phenotyping

Breeding selections were derived from the SH donor parents ‘Yumyeong’, ‘IFF331’, ‘QinWang’ and ‘Hakuto’ back-crossed with different MF accessions and located at the experimental farm of CREA (Forlì, Italy). Fruit phenotype of all selections (including also five accessions with MF texture) was confirmed by measuring post-harvest ethylene emission. Five fruits of each accession were weighted and individually placed into 1,7 l hermetically sealed glass jars. After a 24-h storage at room temperature (22 ± 1 °C), ethylene concentration in the sealed jar head-space was measured using the Ethylene SPY ES100 instrument (Fruit Control Equipment Srl, Milan, Italy) and expressed in ppm of ethylene per kilogram of fresh weight per day (ppm *kg^− 1^ *day^− 1^). Seedlings of two SH segregating F2 populations, BO10040 and BO10039 (42 and 47 individuals, respectively) were grown at the experimental farm ‘Mario Neri’ of ‘CRPV (Centro Ricerca Produzioni Vegetali) - ASTRA’ in Imola (Italy) and managed according to standard cultural practices. The two progenies were obtained from self-pollination of the heterozygous selections ‘BO0501406’ and ‘BO060260 issued from the SH parent ‘D41–62′ crossed with a MF selection [3] and they do not segregate for MF/NMF trait, being the melting parents homozygous for the M allele. For this reason, screening of progenies for the SH trait was carried out directly by monitoring maximum firmness decay (maximum force) during SIII to SIV stage of ripening for at least three seasons.

### Marker validation and candidate variants analyses

Total genomic DNA was extracted from leaf tissues by a modified CTAB protocol and quantified using Qubit (ThermoFisher). Based on previous reference peach transcript annotation V1.0, primers were designed at the flanks of the TC dinucleotide microsatellite on intron I of the ppa008176m gene (*PpYUC11-like*). Forward and reverse primer sequences were 5’-CTATCTGGTATATAAGCTGAAACG-3′ and 5’-CTTGCATGAGGTACTTGGCAC-3′, respectively. The expected amplicon length ranges between 95 base pair for the homozygous TC_20_ repeat to 113 for the TC_29_ [29]. Based on previous knowledge, amplicon size was chosen in order to ensure the discrimination of the TC_20_ repeat fragment from all others, irrespective of the number of repeats (TC_24_, TC_26_ and TC_29_) through a simple agarose gel-based assay. Amplicons were amplified in 10 μl Go-Taq reactions using the following thermal profile: 95 °C for 2 min, 35 cycles of 95 °C for 30 s, 58 °C for 30 s, and 72 °C for 30 s, with a final extension at 72^o^ C for 3 min. PCR products were directly scored through a high-resolution 3% agarose gel-electrophoresis (Metaphor, Lonza, Italy) stained on ethidium-bromide. Following electrophoresis, representative amplicons were excised, purified and directly sequenced to confirm the expected number of repeats.

## Results

### Genome-wide association for the stony hard trait

For GWAS, a panel of 87 accessions was selected based on a well-characterized stony hard (SH) or non-stony hard (non-SH) phenotypes (Additional file 5: Figure S2A). The panel includes traditional Oriental SH accessions (‘Yumyeong’, ‘Jing Yu’, ‘Hua Yu’ and ‘Xia Cui’), breeding selections derived from them, such as ‘189CXIIXLI62’, ‘193QXXVI131’, ‘193QXXVII111’ (from ‘Yumyeong’) and other with unknown origins. Some non-SH accessions, such as ‘Okubo’, ‘Okitsu’ and ‘Hakuto’, but heterozygous for the recessive *hd* locus are also present.

In order to include the effects of population stratification in GWAS, the genetic structure of the panel was inferred by ADMIXTURE software. A value of K = 3 maximizes the predictive accuracy, explaining most of the ancestry among accessions and highlighting the presence of three main clusters (Oriental accessions, breeding-derived and Occidental non-breeding) and several individuals with various degree of admixture, in agreement with the well-known pattern of peach domestication and dispersal (Additional file 5: Figure S2B).

As a proof-of-concept of the statistical power of the GWAS approach, the panel was used to map the non-melting flesh trait (*M/m* locus). The texture phenotypes were coded as binary trait assigning 0–1 to NMF and M accessions, respectively (Additional file 1: Table S1). After adjusting for kinship and population structure, a significant signal was detected on the distal part of chromosome 4 (SNP_IGA_477941, located at 19,898,211 Mbp), both using MLM adjusted for kinship (*p*-value 1.07e-5) and FarmCPU algorithm adjusted for population structure (*p*-value 1.00e-8) (Additional file 6: Figure S3). The associated SNP is located about 800 Kb upstream of the candidate transcript Prupe.4G261900, coding for the *PpendoPGM* protein underlining the MF/NMF trait [16].

**Fig. 1:**
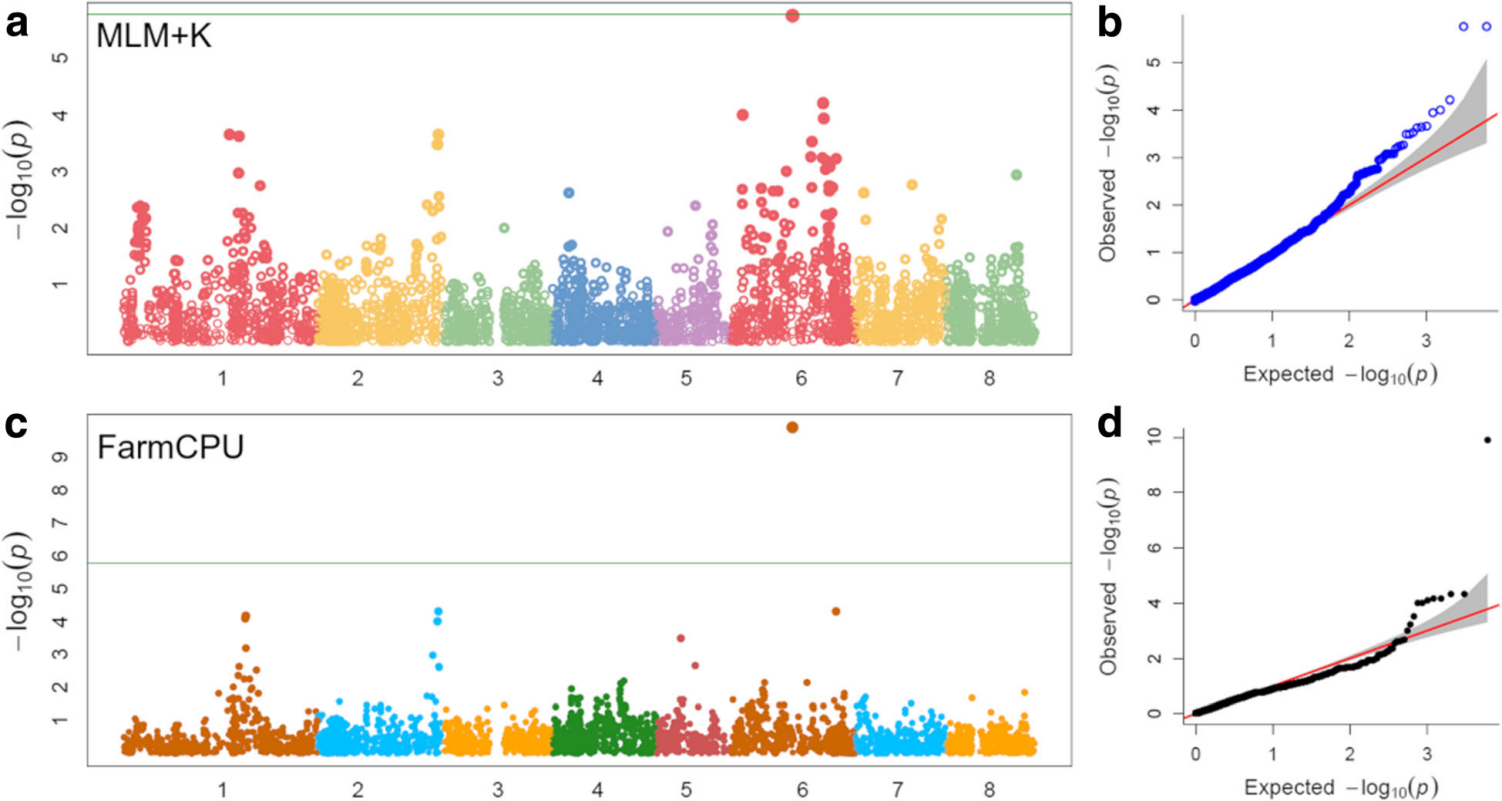
Manhattan plot and QQ-plots of -log_10_*p*-values estimated for stony hard (SH)/non-stony hard (non-SH) trait in a panel of 87 accessions using MLM model adjusted for kinship (**a**, **b**), and FarmCPU algorithm adjusted for population structure (**c**, **d**). Horizontal lines indicate the Bonferroni-adjusted threshold based on the effective number of independent tests

Different statistical models were tested for detecting genome-wide associations for the SH trait (*Hd/hd* locus). The application of mixed models adjusted for kinship and population structure matrices (MLM + K + Q) allowed to detect a highly significant signal on chromosome 6 composed of two closely spaced SNPs (SNP_IGA_538171 and SNP_IGA_538162, with an identical *p*-value of 1.76e-6), close to the Bonferroni threshold (Fig. 1a). As deduced by QQ-plot inspection, the *p*-values distribution suggests a low number of false positive associations (Fig. 1b). FarmCPU algorithm improves the significance of the most associated marker, assigning a *p*-value of 1.25e-10 to SNP_IGA_538171 and further reducing background inflation (Fig. 1c, d). The marker is located at 15,228,028 Mbp in a pericentromeric region of chromosome 6. In the analysed panel, the minor allele at the SNP_IGA_538171 locus showed a frequency of 0.12, being homozygous in 8 out of 12 SH genotypes, and heterozygous in ‘Hakuto’, ‘Okubo’ and ‘Okitsu’, known for bearing the recessive *hd* allele.

### Gene mining and DEG analysis

**Fig. 2:**
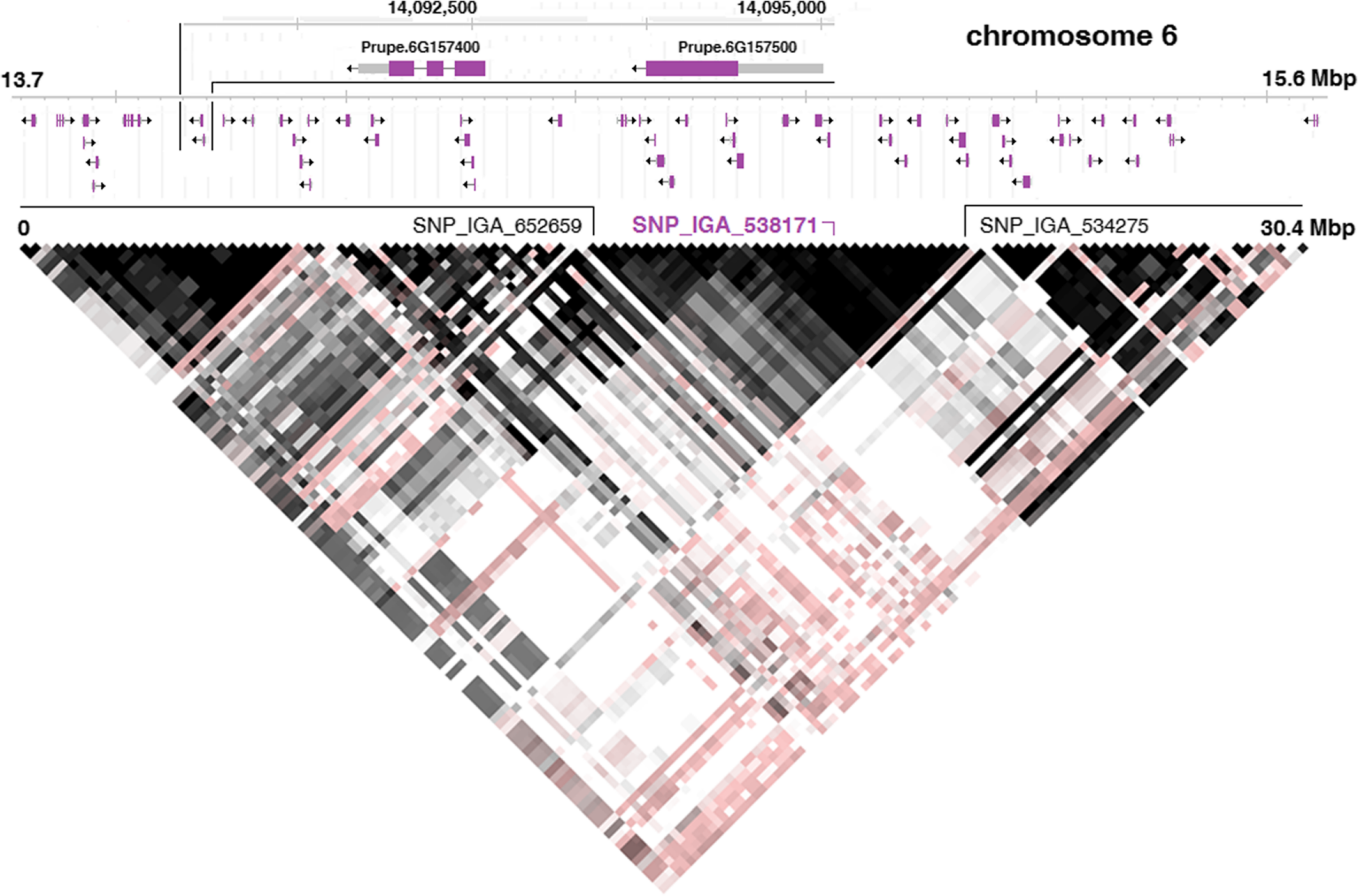
Linkage disequilibrium pattern and annotated transcripts in peach reference genome around the most associated SNP_IGA_538171 (in violet). The *hd* locus is delimited by SNP_IGA_652659 (13,743,178 bp) and SNP_IGA_534275 (15,609,595 bp)

Linkage disequilibrium (LD) analysis of the regions surrounding the SNP_IGA_538171 tagged by the array revealed an extended LD block (D’ values higher than 0.8), which delimits the identified *hd* locus to a region of about 1.9 Mb in physical size, roughly comprised between SNP_IGA_652659 (13,743,178 bp) and SNP_IGA_534275 (15,609,595 bp) (Fig. 2). The observed LD pattern was expected considering the low recombination rate in chromosomal regions near the centromere [40]. The gene inventory of this region comprises 69 transcripts, of which 52 having functional homologies in Arabidopsis or other plant species (Additional file 7: Table S4). The list includes the candidate gene *PpYUC11-like* (ppa008176m according to the v1.0 nomenclature), predicted to encode two separated transcripts on the reverse strand: Prupe.6G157400 (from II to IV exon) and Prupe.6G157500 (I exon), both spanning the region from 14,091,690 to 14,095,013 base pair, and located at about 1.1 Mb from the most significantly associated marker (SNP_IGA_538171) (Fig. 2). Apart from this gene, the candidate list includes other transcripts, albeit with no previous validated function in fruit development or ripening: a putative cellulose synthase *CESA6-like* (Prupe.6G161300) and two cinnamoyl alcohol dehydrogenase *CAD9-like* (Prupe.6G161800 and Prupe.6G162000), putatively involved in cell-wall metabolism, as well as several putative kinases (among which an RLK1 and a CLAVATA1-like proteins) having an array of biological functions.

**Fig. 3:**
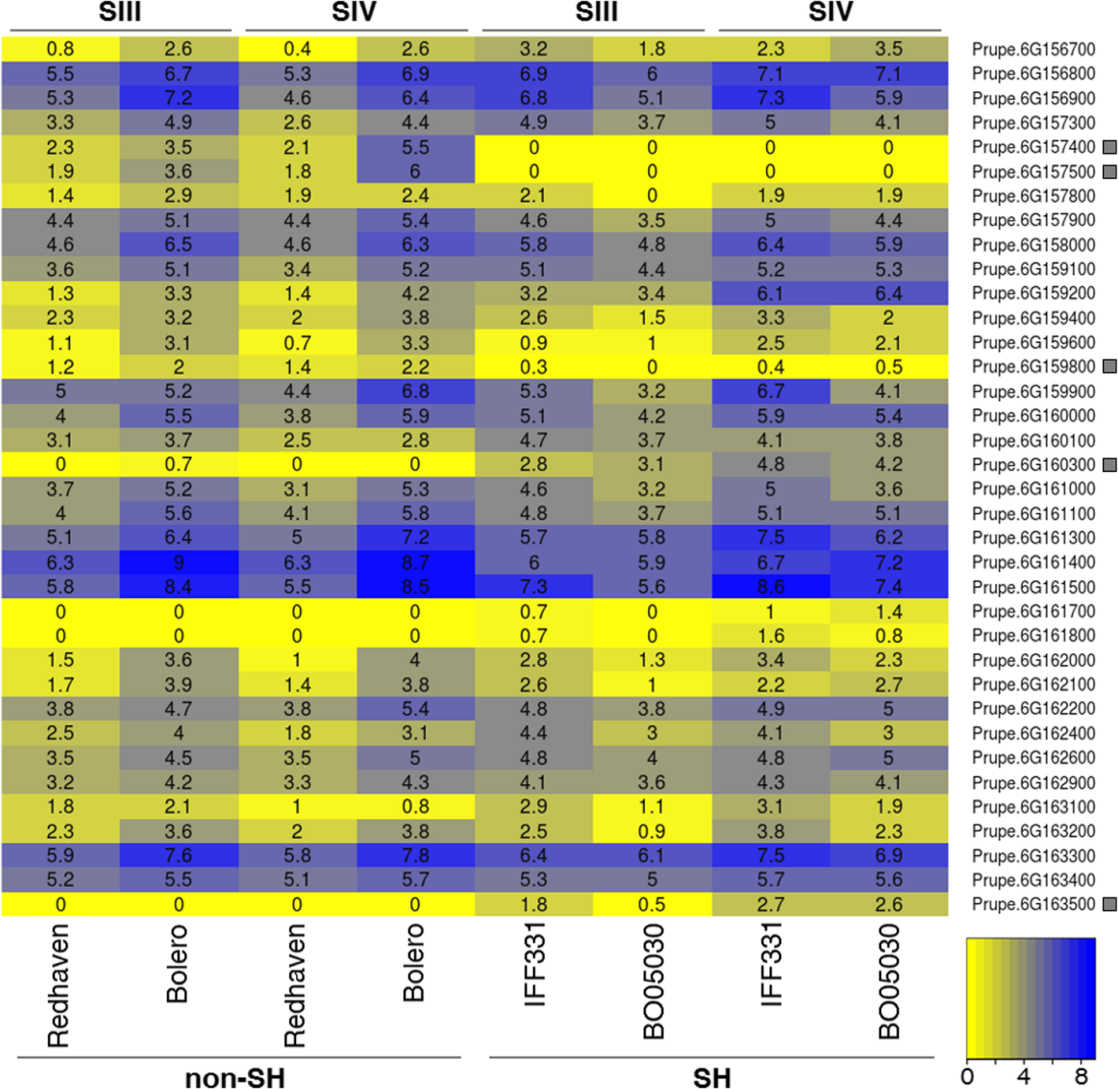
Transcriptome analysis for the identification of candidate genes for SH trait within the *hd* locus. Heat map of transcripts abundance (expressed as log_2_RKPM) in two non-SH (melting flesh), ‘Bolero’ and ‘Redhaven’, and two SH, ‘IFF331’ and ‘BO05030081’ accessions at stages III and IV of fruit development. Black squares indicate differentially expressed genes from the comparison of SH vs non-SH accessions

**Fig. 4:**
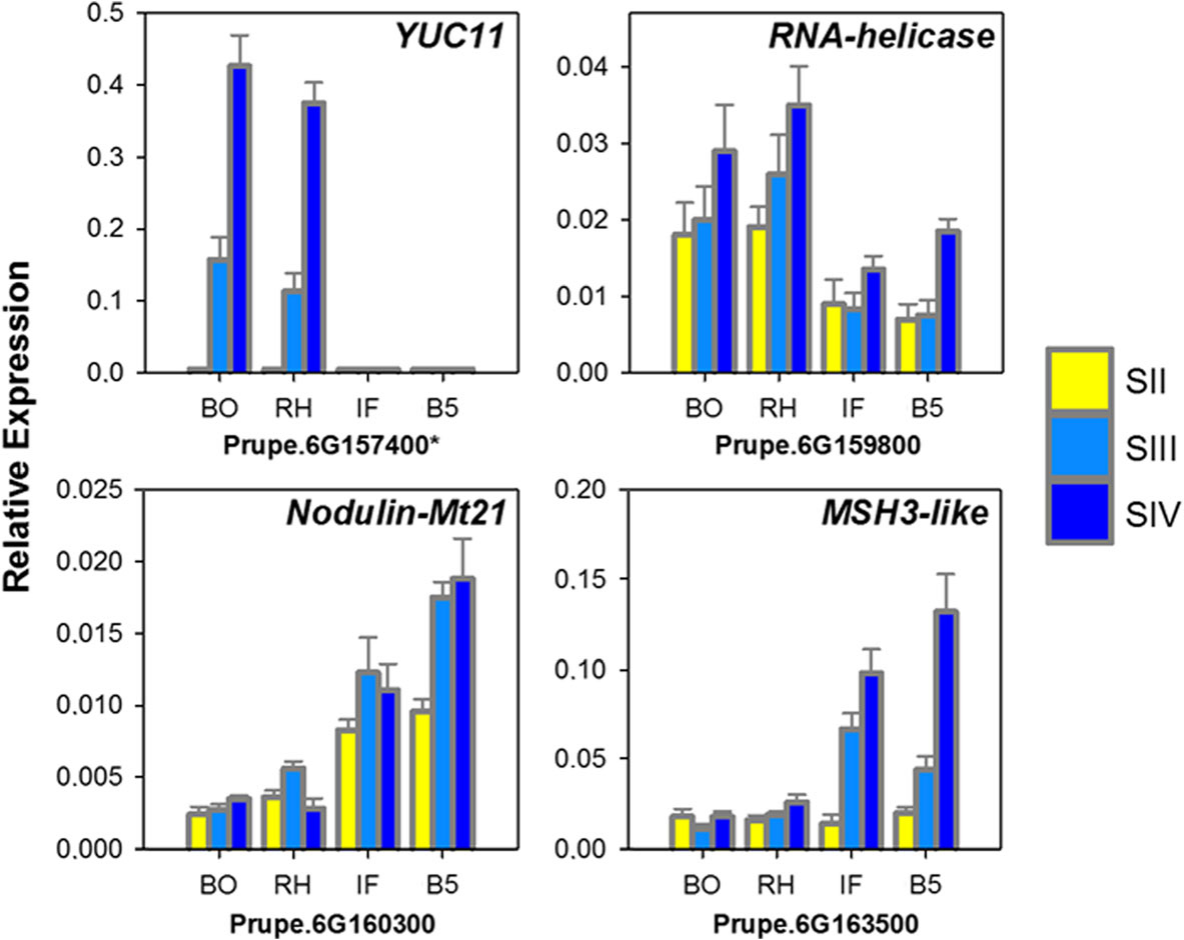
Real-time PCR analysis of four selected differentially expressed transcripts within *hd* locus in two non-SH (melting flesh), ‘Bolero’ (BO) and ‘Redhaven’ (R), and two SH, ‘IFF331’ (IF) and ‘BO05030081’ (B5) accessions at stages II, III and IV of fruit development

An overview of the expression patterns of annotated transcripts within the selected interval was obtained from fruit transcriptome data. Genes differentially expressed between SH and non-SH fruits were identified through the comparison of two melting flesh, ‘Bolero’ and ‘Redhaven’ and two SH accessions, ‘BO05030081’ (a selection issued from 'D41–62') and ‘IFF331’, along SIII and SIV stages of ripening, when the two types of texture begin to differentiate. As depicted by the heat-map (based on log_2_RKPM values), a few transcripts present within *hd* locus showed a differential expression pattern between the two texture types (Fig. 3). The most obvious was the lack of Prupe.6G157400.1 and Prupe.6G157500.1 (both coding for *PpYUC11-like*) expression in SH fruits, in contrast to the melting ones, where it was up-regulated at ripening (Fig. 4). Other transcripts showed a different abundance between MF and SH, such as Prupe.6G159800 (coding for an DHX16-like RNA helicase) showing the same trend and magnitude of *PpYUC11-like*; Prupe.6G160300 (coding for a *nodulin Mt21-like* gene) and Prupe.6G163500 (similar to DNA mismatch repair protein MSH3) only expressed in SH fruits (Fig. 4). Outside the *hd* locus, the list of differentially expressed genes includes other important and well-characterized ripening-related transcripts, such as *ACS1* (ACC synthase), a key enzyme of ethylene biosynthesis, *end*o-PGM, the main polygalacturonase regulating the melting process, and a GH3.3-like, an auxin-conjugating enzyme, all down-regulated in SH fruits (data not shown). The same trend characterized also several genes implicated in auxin metabolism, perception and signalling pathways.

### Genomic variations at *hd* locus

Further insights about the associations between genomic variations at the *hd* locus and SH phenotype were obtained by inspecting high-coverage re-sequencing data of the SH accession ‘D41–62’, in comparison with whole-genome alignment of several SH and non-SH (MF, melting flesh; NMF, non-melting flesh) accessions with different genetic origins. According to the genetic inheritance of SH trait, putatively associated variants had to be: i) homozygous and shared between SH genotypes; ii) heterozygous in non-SH genotypes carry on the recessive *hd* allele (e.g. ‘Okubo’ and ‘Okitsu’); iii) absent in non-SH genotypes. In line with the multiple evidences supporting the *PpYUC11-like* gene as the causative locus of SH phenotype, several polymorphisms conserved among SH accessions were identified in nearby regions. In addition to the already reported partial deletion of TC repeat within the intron microsatellite, other interesting variants were represented by two INDELs (a 26-bp insertion and a 20-bp deletion) respectively located in the promoter region (at 14,095,453 bp) and 5’UTR (at 14,093,515 bp) of the gene. However, the 5’UTR variant is only present in the SH accession ‘Jing Yu’ but not in ‘D41–62’ or ‘Yumyeong’, whereas the upstream INDEL was identified in other non-SH accessions (Additional file 8: File S1). Apart from *PpYUC11-like* gene, a discrete number of polymorphisms (mainly SNPs) were also identified in upstream and/or downstream regulatory regions of differentially expressed genes Prupe.6G159800 and Prupe.6G160300 (Additional file 8: File S1). Most of these variants are in perfect linkage with the TC_20_ allele within *PpYUC11-like* intron. Furthermore, putative SH-specific high-impact variants were identified in Prupe.6G158800 (a frame-shift TGG > T insertion at position 14,387,591) and Prupe.6G159300 (loss of stop codon, G > A substitution at 14,585,488), although their involvement in SH trait can be excluded, being these genes not expressed in fruit tissues.

### Variant-trait association

**Fig. 5:**
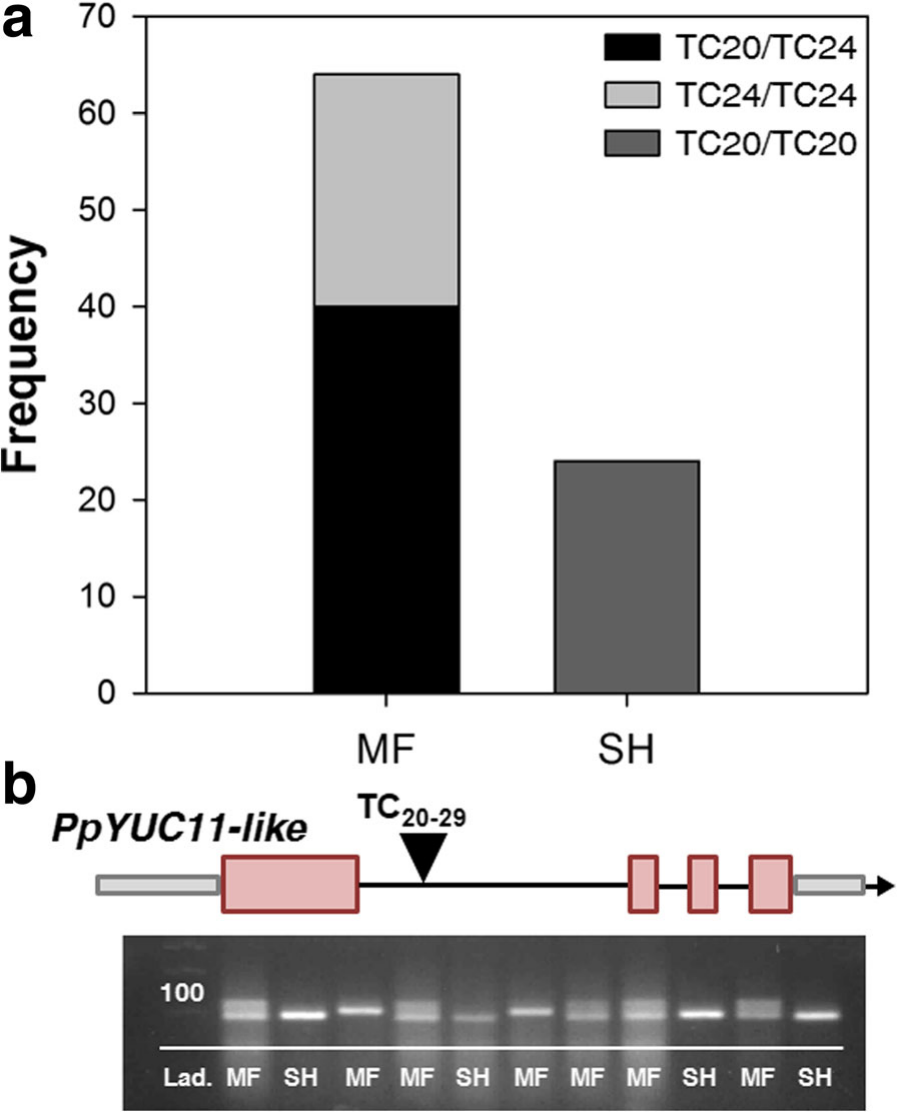
Frequency of melting flesh (MF) and stony hard (SH) phenotypes in two combined F_2_ segregating progenies and co-segregation with TC_20_ and TC_24_ alleles within *PpYUC11-like* intron. An example of electrophoresis-gel based genotyping of TC microsatellite variants

**Fig. 6:**
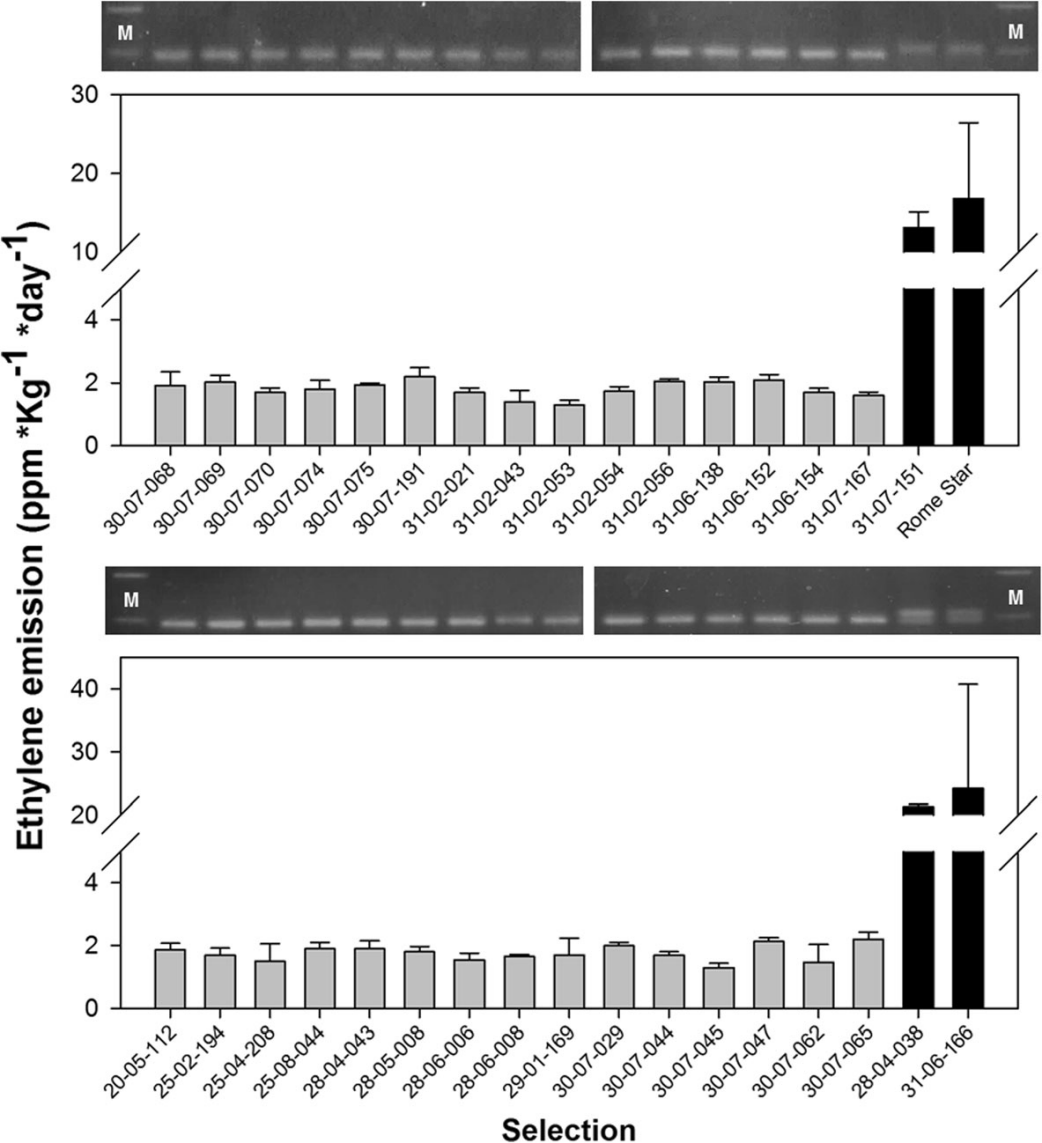
Ethylene level at harvest in 30 SH and 3 non-SH (melting flesh) breeding selections, and ‘Rome Star’ accession. The TC_20_ allele within *PpYUC11-like* intron microsatellite resulted homozygous in all SH selections

Co-localization of GWAS signals, together with transcriptome profiling and genome sequencing, added multiple layers of evidence to the candidate gene *PpYUC11-like*. Nonetheless, a further step of validation was provided through the evaluation of variant-trait association in bi-parental F_2_ segregating populations and advanced breeding selections. Two segregating progenies were considered: a total of 24 individuals out of 89 were phenotyped as SH based on firmness measurements (13 out of 42 and 11 out of 47, respectively for BO10040 and BO10039 progenies) (Fig. 5a and Additional file 9: Table S5). The marker at intron TC microsatellite locus segregated in about 1:2:1 ratio in both progenies (chi-square of 1.21 and 0.80, respectively for BO10039 and BO10040) was perfectly associated with SH trait (Fig. 5b). Also, the percentage of observed SH individuals agreed with the expected 3:1 segregation pattern of non-SH vs SH (chi-square of 0.06 and 0.79, respectively for the two progenies). Parents of both progenies were heterozygous for the TC_20_/TC_24_ (*Hd/hd*) alleles, all SH individuals were homozygous for the TC_20_ allele (*hd/hd*) and MF individuals homozygous for the TC_24_ allele (*Hd/Hd*) or heterozygous (Additional file 9: Table S5). Advanced selections for SH trait were characterized by barely detectable post-harvest ethylene emission, steadily below the concentration of 3 ppm, and all turned out to be homozygous for the TC_20_ allele (*hd/hd*); in contrast ethylene production ranged from 13 to 94 ppm *Kg^− 1^ *day^− 1^ within the control MF accessions, which were either homozygous or heterozygous for the TC_24_ allele (*Hd/Hd or Hd/hd*) (Table 1 and Fig. 6).

**Table 1:** Cross parents of selections, maturity degree (I_AD_ index) and ethylene level at harvest in 33 breeding selections and 2 accessions (‘Rome Star’ and ‘Regina Bianca’) used in this study

Cross parents	Selection	Fruit type	Fresh weight (g)	I_AD_	C_2_H_4_ (ppm*Kg^−1^*day^− 1^)	*hd* locus (allele size)
*Stony hard texture*						
IFF331 op	20–05-112	pws	151.9 ± 23.5	0.27 ± 0.06	1.87 ± 0.21	97/97
[(Hakuto x Snowqueen) op] x op	25–02-194	nws	175.0 ± 31.9	0.61 ± 0.22	1.70 ± 0.22	97/97
Yumyeong x [IFF631 x (O’Henry x Snowqueen)]	25–04-208	pws	202.9 ± 19.7	0.27 ± 0.01	1.50 ± 0.56	97/97
(O’Henry x Yumyeong) sp	25–08-044	pys	164.8 ± 15.2	0.27 ± 0.08	1.90 ± 0.20	97/97
(O’Henry x Yumyeong) sp	28–04-043	pwa	195.5 ± 21.0	0.57 ± 0.12	1.90 ± 0.25	97/97
(O’Henry x Yumyeong) sp	28–05-008	pws	209.6 ± 22.0	0.10 ± 0.02	1.80 ± 0.17	97/97
[Yumyeong x (Botto x Royal Glory))] x op	28–06-006	pws	168.8 ± 24.7	0.02 ± 0.02	1.55 ± 0.21	97/97
[Yumeyong x (Botto x Royal Glory))] x op	28–06-008	pws	156.2 ± 12.8	0.02 ± 0.01	1.65 ± 0.07	97/97
(IFF331 x Big Top) x op	29–01-169	pys	191.0 ± 20.2	0.27 ± 0.24	1.70 ± 0.53	97/97
Quing Wa x FRF1373	30–07-029	pws	117.2 ± 10.2	0.27 ± 0.05	2.00 ± 0.10	97/97
FRF1681 (sdg. Ma 25–02-194) x op	30–07-044	nwa	80.9 ± 15.0	1.12 ± 0.31	1.70 ± 0.10	97/97
FRF1681 (sdg. Ma 25–02-194) x op	30–07-045	nwa	103.8 ± 20.1	0.37 ± 0.07	1.30 ± 0.14	97/97
FRF1681 (sdg. Ma 25–02-194) x op	30–07-047	nws	75.5 ± 16.0	0.27 ± 0.18	2.13 ± 0.12	97/97
FRF1681 (sdg. Ma 25–02-194) x op	30–07-062	nwa	116.5 ± 7.6	0.27 ± 0.16	1.47 ± 0.57	97/97
FRF1681 (sdg. Ma 25–02-194) x op	30–07-065	nws	57.1 ± 9.7	0.15 ± 0.03	2.20 ± 0.23	97/97
FRF1681 (sdg. Ma 25–02-194) x op	30–07-068	nws	128.2 ± 22.9	0.57 ± 0.12	1.93 ± 0.43	97/97
FRF1681 (sdg. Ma 25–02-194) x op	30–07-069	nwa	74.9 ± 10.6	0.27 ± 0.09	2.03 ± 0.21	97/97
FRF1681 (sdg. Ma 25–02-194) x op	30–07-070	nwa	111.3 ± 15.4	0.20 ± 0.10	1.70 ± 0.14	97/97
FRF1681 (sdg. Ma 25–02-194) x op	30–07-074	nwa	105.7 ± 18.8	0.08 ± 0.00	1.80 ± 0.28	97/97
FRF1681 (sdg. Ma 25–02-194) x op	30–07-075	nwa	95.4 ± 5.5	0.27 ± 0.02	1.93 ± 0.06	97/97
Hakuto x FRF1373	30–07-191	pws	100.8 ± 10.6	0.08 ± 0.07	2.20 ± 0.28	97/97
FRF1679 x FRF1681	31–02-021	nys	151.2 ± 0.5	0.52 ± 0.10	1.70 ± 0.14	97/97
FRF1679 x FRF1681	31–02-043	nys	107.0 ± 11.6	0.27 ± 0.06	1.40 ± 0.36	97/97
FRF1679 x FRF1681	31–02-053	nws	76.3 ± 15.1	0.08 ± 0.02	1.30 ± 0.15	97/97
FRF1679 x FRF1681	31–02-054	pys	125.5 ± 7.3	0.27 ± 0.02	1.73 ± 0.15	97/97
FRF1679 x FRF1681	31–02-056	nws	126.6 ± 15.2	0.17 ± 0.08	2.05 ± 0.07	97/97
FRF1679 x FRF1681	31–06-138	pys	112.4 ± 19.0	0.27 ± 0.06	2.03 ± 0.15	97/97
FRF1679 x FRF1681	31–06-152	pys	150.6 ± 22.1	0.17 ± 0.04	2.10 ± 0.17	97/97
FRF1679 x FRF1681	31–06-154	pws	162.3 ± 18.2	0.16 ± 0.03	1.70 ± 0.13	97/97
FRF1681 x FRF1678 (sdg. Ma 25–02-187)	31–07-167	nys	80.2 ± 10.5	0.23 ± 0.05	1.60 ± 0.11	97/97
*Melting Flesh texture*						
(Red Star x Alitop) x Romagna 3000	31–07-151	pwa	79.5 ± 9.7	0.23 ± 0.05	13.10 ± 2.02	104/104
FRF1207 (Ma 16–05-089) x (Royal Prince x Yoshihime)	31–06-166	nys	108.5 ± 22.7	0.27 ± 0.09	24.20 ± 16.54	104/104
(O’Henry x Yumyeong) x op	28–04-038	pwa	253.8 ± 25.6	0.38 ± 0.25	21.35 ± 0.35	104/97
Regina Bianca	–	pwa	125.4 ± 11.1	0.11 ± 0.02	94.50 ± 0.71	104/104
Rome Star	–	pya	149.2 ± 15.7	0.27 ± 0.04	16.83 ± 9.62	104/104

Main fruit quality characteristics are also indicated (p/n peach/nectarine; y/w yello*w*/white flesh color; a/s acid/low acid fruit taste) along with the allelic status at *YUC11-like* intron TC microsatellite

## Discussion

Understanding the genetic basis of peach textural characteristic is an essential step for the exploitation of the natural phenotypic variability and as a model for other soft fruit species. Among the various types of peach textures, SH type has received remarkable interest, both at biological and applicative research levels [9, 26]. A series of studies during the last decade have clarified the physiological background of the SH mutation, associated to a deficiency in fruit IAA production, the hormone which orchestrates the peach ripening processes through the activation of system 2 ethylene pathway [4, 36]. Recently, a polymorphic TC repeat in the first intron of *a PpYUC11-*like gene has been proposed as the candidate determinant of SH trait, in the light of molecular and functional evidences [29].

In the present work, the genetic architecture of the SH trait has been clarified through genome-wide association and linkage disequilibrium analyses in a panel of accessions with different textures, delimiting the *hd* locus to an interval of about 1.9 Mbp in the centromeric regions of chromosome 6. Gene mining across the target genomic interval, coupled with fruit transcriptome data from MF and SH accessions, allowed to prioritize a limited number of differentially regulated transcripts, among which *PpYUC11-like* and a few others with no previous evidence of a function in ripening-related phenomena. Comparison of resequencing data of the SH peach ‘D41–62’ with several SH and non-SH, MF and NMF accessions of different genetic origins, provided an accurate overview of genomic variation present at *hd* locus. This layer of information was essential for identifying variants putatively affecting regulatory and/or coding regions. Sequencing data clearly prioritized the intron TC_20_ microsatellite allele on *PpYUC11-like,* although a discrete number of SH-specific variants were also identified in regulatory regions of other differentially expressed genes within the mapped interval. While the involvement of such variants in SH trait cannot be fully excluded, they most probably reflect strong local LD around the SH locus, arising from the low recombination events in pericentromeric regions [40]. The segregation pattern of the TC_20_ allelic variant was evaluated in a broader genetic background, consisting of several advanced selections and two bi-parental progenies derived from the SH ‘D41–62’ parent. The perfect co-segregation adds a further level of validation, which, together with genomic, physiological and molecular evidences, allows to confirm with high confidence the role of allelic variations at *PpYUC11-like* as the genetic determinant of the SH phenotype.

Although ultimate proof of the causal link between the candidate gene and the SH trait may require genetic engineering approaches, the body of evidence around *PpYUC11-like* opens interesting perspectives. At biological level, a key role for auxin in the regulation of peach ripening has been long demonstrated, as well as its intense interplay with ethylene [37]. The SH mutation is a first example of the role of auxin in shaping fruit textural characteristics in peach. However, little is known about the phenotypic variability for fruit auxin content at peach population level, nor its effects on melting processes and texture changes outside the SH background. A certain genetic variability has been already reported within the intron TC microsatellite, particularly in accessions derived from Oriental germplasm [29]. Considering the broad quantitative variation within melting flesh texture type, allelic variability within *PpYUC11-like* or in other genes of the YUCCA family (such as the ripening-related *PpYUC10-like)*, may represent interesting targets for future studies in peach as well as in other soft fruit species.

## Conclusions

In this study, we provide a multi-level validation of the genetic control of the SH trait through the integration of genome-wide association mapping, transcriptome analysis and whole-genome resequencing data for SH and non-SH accessions, and marker-trait association in a panel of advanced breeding selections and segregating progenies. Collectively, data confirm with high confidence the role of allelic variation at *PpYUC11-like* locus as the genetic determinant of the SH trait, opening interesting perspectives at both biological and applied research level.

## Additional files

Additional file 1: **Table S1.** List of analyzed peach accessions and respective texture phenotype. (DOCX 19 kb)

Additional file 2: **Figure S1.** Evolution of flesh firmness during ripening of four peach accessions with a stony hard (‘BO05030081’ and ‘IFF331’) or non-SH/MF (‘Bolero’, ‘Redhaven’) texture, as measured through a penetration-based test. (BMP 4181 kb)

Additional file 3: **Table S2.** List of primers used for quantitative PCR analyses. (DOCX 12 kb)

Additional file 4: **Table S3.** SRA accession number of assembled Illumina Whole-Genome libraries. (DOCX 13 kb)

Additional file 5: **Figure S2.** A) Histogram summarizing the frequency of stony hard (SH)/non-stony hard (non-SH) and melting (MF)/non-melting (NMF) phenotypes in a panel of 87 accessions used for GWAS; B) Genetic structure plot of the analysed panel for the optimal number of a priori genetic clusters (K = 3), with ancestry proportion on the Y-axis. The red, green and light blue bars indicate the subpopulations I (breeding-derived), subpopulation II (Occidental, non-breeding) and subpopulation III (Oriental origins), respectively. (BMP 1255 kb)

Additional file 6: **Figure S3.** Manhattan and quantile-quantile (QQ) plots of the -log_10_*p*-values estimated for MF/NMF trait in a panel of 87 accessions using FarmCPU algorithm adjusted for population structure (left panel). Red horizontal indicates significant SNPs passing the Bonferroni-adjusted threshold based on the effective number of independent tests. (BMP 1220 kb)

Additional file 7: **Table S4.** List of candidate genes identified on the chromosome 6 region comprised between SNP_IGA_652659 (13,743,178 bp) and SNP_IGA_534275 (15,609,595 bp). (DOCX 17 kb)

Additional file 8: **File S1.** Full list of variants annotations and effects (as calculated with SNPEff tool) from whole genome sequencing assembly of 19 accessions within the *hd* locus on chromosome 6. Candidate variant on PpYUC11-like gene is highlighted in black; variants associated to DEGs in yellow; high-impact variants predicted by SNPEff in red. See text for variants filtering criteria. (XLSX 41 kb)

Additional file 9: **Table S5.** Phenotypic evaluation for SH trait and allelic status at YUC11 TC microsatellites of the two F_2_ segregating progenies BO10040 and BO10039 (issued from the SH parent ‘D4162’). (DOCX 16 kb)

## Abbreviations

GWAS: Genome-wide association
LD: Linkage disequilibrium
MF: Melting flesh
MLM: Mixed linear model
NMF: Non-melting flesh
SH: Stony hard
WGS: Whole-genome sequence
