# Supplementary material for: Integrative genomics approaches validate PpYUC11-like as candidate gene for the stony hard trait in peach (P. persica L. Batsch)
Source: BMC Plant Biol. 2018 May 18;18:88. doi: 10.1186/s12870-018-1293-6 (PMC5960097; doi:10.1186/s12870-018-1293-6)
Supplement: Supplementary file 1 — Table S1. List of analyzed peach accessions and respective texture phenotype. (DOCX 19 kb) [file 12870_2018_1293_MOESM1_ESM.docx]

| **Accession** | **Cross parents** | **Fruit Texture** | **SH/non-SH**  **(0 - 1)** | **NMF/MF**  **(0 - 1)** |
| --- | --- | --- | --- | --- |
| 189CXIIXLI62 | Yumyeong op. | Stony hard | 0 | 1 |
| 193QXXVI131 | Yumyeong op. | Stony hard | 0 | 1 |
| 193QXXVII111 | Yumyeong op. | Stony hard | 0 | 1 |
| 194RXXIII43 | - | Stony hard | 0 | 1 |
| AFRA T.15/9 | - | Melting | 1 | 1 |
| ALEXA | breeding, Italy | Melting | 1 | 1 |
| ALICECOL | breeding, Italy | Non-Melting | 1 | 0 |
| ALIPERSIE | breeding, Italy | Melting | 1 | 1 |
| ALMA | breeding, Italy | Melting | 1 | 1 |
| AMBRA | Stark Red Gold x Mayfire | Melting | 1 | 1 |
| ANDROSS | Dix5A1 x Fortuna | Non-Melting | 1 | 0 |
| AURORA | Chinese Cling x MaryChoice | Non-Melting | 1 | 0 |
| A. MARZOCCHELLA | Vesuvio mut. | Non-Melting | 1 | 0 |
| AUTUMN GRAND | Late Le Grand x GoldKing op. | Melting | 1 | 1 |
| AZURINA | Red Robin sp. | Melting | 1 | 1 |
| BEIJING | unknown, China | Melting | 1 | 1 |
| BELLA DI CESENA | unknown, Italy | Melting | 1 | 1 |
| BIG TOP | breeding, USA | Melting | 1 | 1 |
| BLAZING STAR | breeding, USA | Melting | 1 | 1 |
| BLUSHING STAR | breeding, USA | Melting | 1 | 1 |
| BOLERO | Cresthaven x Flamecrest | Melting | 1 | 1 |
| BONIA | breeding, Italy | Non-Melting | 1 | 0 |
| BORGIA | Maycrest op. | Non-Melting | 1 | 0 |
| BOUNTY | B60324 x B64237 | Melting | 1 | 1 |
| CAPUCCI18 | Sant'Anna Balducci op. | Melting | 1 | 1 |
| CHIMARRITA | Babcock x Flordabelle | Melting | 1 | 1 |
| CONTENDER | Winblo x NC64 | Melting | 1 | 1 |
| CRISTINA | breeding, USA | Melting | 1 | 1 |
| D41-62 | unknown, China | Stony hard | 0 | 1 |
| DA TIAN TAO | unknown, China | Melting | 1 | 1 |
| DIXIRED | Halehaven sp. | Melting | 1 | 1 |
| EARLIRICH | breeding, USA | Melting | 1 | 1 |
| EARLY TOP | Redtop mut. | Melting | 1 | 1 |
| EARLY ZEE | breeding, USA | Melting | 1 | 1 |
| ELBERTITA | Elberta dwarf op. | Melting | 1 | 1 |
| ELEGANT LADY | Early O'Henry x July Lady | Melting | 1 | 1 |
| FEI CHENG BAI LI | unknown, China | Non-Melting | 1 | 0 |
| GLOHAVEN | J.H. Hale op. x Kalhaven | Melting | 1 | 1 |
| HAKUTO | Chinese Cling op. | Melting | 1 | 1 |
| HONEY BLAZE | breeding, USA | Melting | 1 | 1 |
| HONEY GLO | breeding, USA | Melting | 1 | 1 |
| HONEY KIST | breeding, USA | Melting | 1 | 1 |
| HUA YU | unknown, China | Stony hard | 0 | 1 |
| IF691 | unknown, Italy | Non-Melting | 1 | 0 |
| IFF331 | Hakuto x NJ256 | Stony hard | 0 | 1 |
| IONIA | Vivian x Federica | Non-Melting | 1 | 0 |
| J.H. HALE | unknown, USA | Melting | 1 | 1 |
| JING YU | Okubo x Okitsu | Stony hard | 0 | 1 |
| KAMARAT | unknown, Italy | Melting | 1 | 1 |
| KAWEAH | breeding, USA | Melting | 1 | 1 |
| LUCREZIA | Maycrest op. | Melting | 1 | 1 |
| MAGIQUE | breeding, France | Melting | 1 | 1 |
| MARUJA | unknown, Spain | Non-Melting | 1 | 0 |
| MAURA | breeding, USA | Melting | 1 | 1 |
| MAYCREST | Springcrest mut. | Melting | 1 | 1 |
| MAYFIRE | Armking op. | Melting | 1 | 1 |
| MERRIL GEM FREE | July Elberta x Merril Gem | Melting | 1 | 1 |
| NADIA | breeding, Italy | Melting | 1 | 1 |
| NJ WEEPING | unknown, USA | Melting | 1 | 1 |
| NJ307 | unknown, USA | Stony hard | 0 | 1 |
| OKITSU | Precoce de Croncels x Lord Napier | Melting | 1 | 1 |
| OKUBO | unknown, Japan | Melting | 1 | 1 |
| OURO IAPAR | unknown, Brazil | Melting | 1 | 1 |
| PIERI81 | Elberta x Sant'Anna | Melting | 1 | 1 |
| REDHAVEN | Halehaven x Kalhaven | Melting | 1 | 1 |
| RICH LADY | Amparo op. | Melting | 1 | 1 |
| RISING STAR | Newhaven x Jim Dandee | Melting | 1 | 1 |
| RITA STAR | (Aurelio Grand x peach) op. | Melting | 1 | 1 |
| ROSA DARDI | unknown, Italy | Melting | 1 | 1 |
| ROYAL GLORY | MayGrand op. | Melting | 1 | 1 |
| RUBIA | breeding, Italy | Melting | 1 | 1 |
| RUBYRICH | breeding, USA | Melting | 1 | 1 |
| S5898:128 | Sant'Anna mut. | Melting | 1 | 1 |
| S6699 | breeding, Italy | Melting | 1 | 1 |
| SOLEADA | breeding, Italy | Melting | 1 | 1 |
| SPRING BABY | P51-2 x P51-103 | Non-Melting | 1 | 0 |
| STONEY HARD | Koyohakuto x Okubo | Stony hard | 0 | 1 |
| SUPEACH FOUR | Flavorcrest x Springcrest | Melting | 1 | 1 |
| TURQUOISE | breeding, France | Melting | 1 | 1 |
| VEECLING | Babygold 6 op. | Non-Melting | 1 | 0 |
| VENUS | Stark Redgold x Flamekist | Melting | 1 | 1 |
| VITTORIO EMANUELE | unknown, Italy | Melting | 1 | 1 |
| VISTARICH | breeding, USA | Melting | 1 | 1 |
| XIA CUI | unknown, China | Stony hard | 0 | 1 |
| XIA HUI | unknown, China | Melting | 1 | 1 |
| YANG HUANG | unknown, China | Melting | 1 | 1 |
| YUMYEONG | Yamato-Wase x Nunome-Wase | Stony hard | 0 | 1 |
