# Supplementary figures and images for: Integrative genomics approaches validate PpYUC11-like as candidate gene for the stony hard trait in peach (P. persica L. Batsch)
Source: BMC Plant Biol. 2018 May 18;18:88. doi: 10.1186/s12870-018-1293-6 (PMC5960097; doi:10.1186/s12870-018-1293-6)

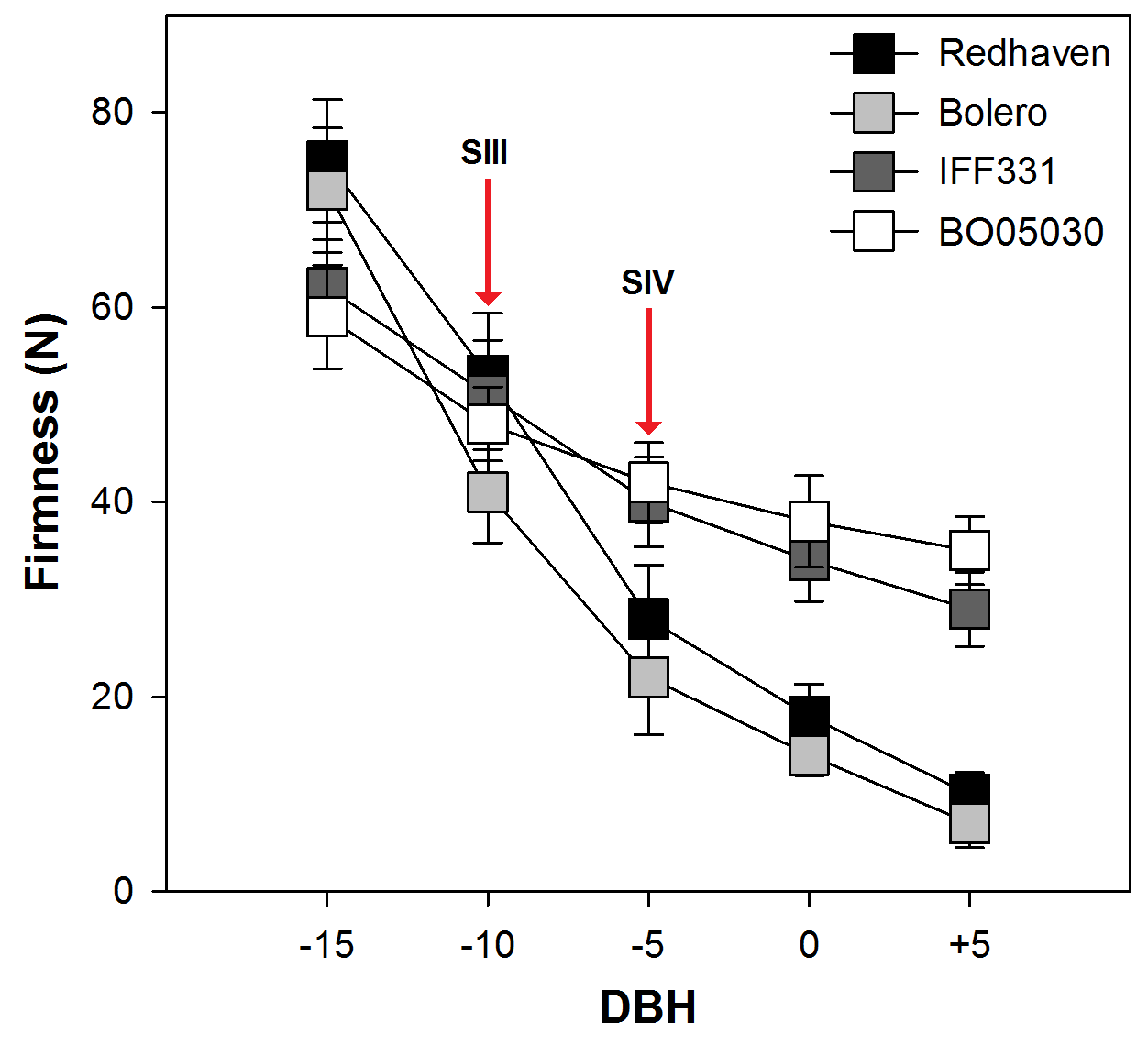

Supplement: Supplementary file 2 — Figure S1. Evolution of flesh firmness during ripening of four peach accessions with a stony hard (‘BO05030081’ and ‘IFF331’) or non-SH/MF (‘Bolero’, ‘Redhaven’) texture, as measured through a penetration-based test. (BMP 4181 kb) [file 12870_2018_1293_MOESM2_ESM.bmp]

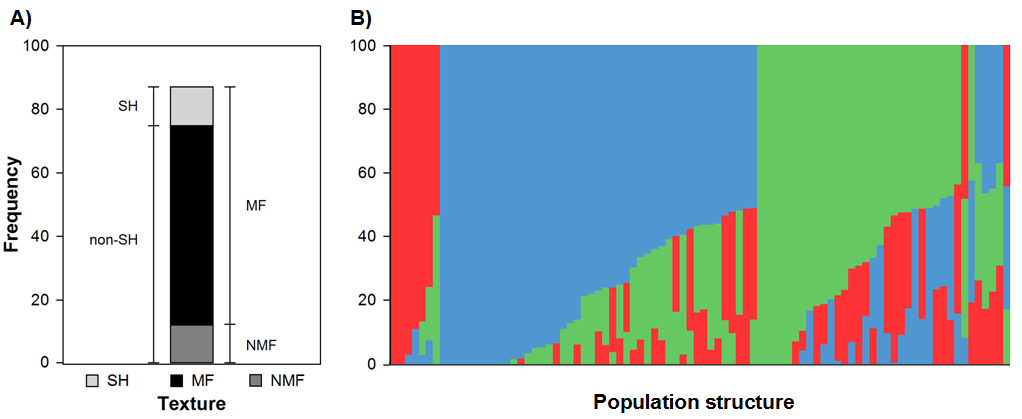

Supplement: Supplementary file 5 — Figure S2. A) Histogram summarizing the frequency of stony hard (SH)/non-stony hard (non-SH) and melting (MF)/non-melting (NMF) phenotypes in a panel of 87 accessions used for GWAS; B) Genetic structure plot of the analysed panel for the optimal number of a priori genetic clusters (K = 3), with ancestry proportion on the Y-axis. The red, green and light blue bars indicate the subpopulations I (breeding-derived), subpopulation II (Occidental, non-breeding) and subpopulation III (Oriental origins), respectively. (BMP 1255 kb) [file 12870_2018_1293_MOESM5_ESM.bmp]

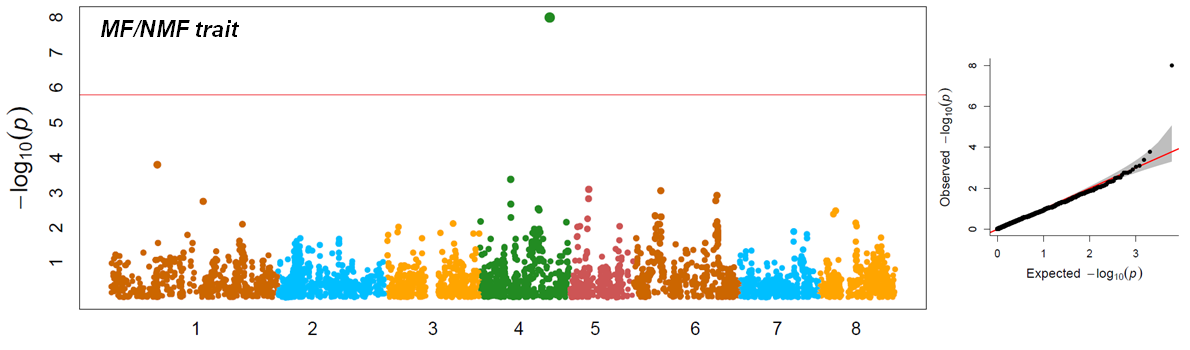

Supplement: Supplementary file 6 — Figure S3. Manhattan and quantile-quantile (QQ) plots of the -log10p-values estimated for MF/NMF trait in a panel of 87 accessions using FarmCPU algorithm adjusted for population structure (left panel). Red horizontal indicates significant SNPs passing the Bonferroni-adjusted threshold based on the effective number of independent tests. (BMP 1220 kb) [file 12870_2018_1293_MOESM6_ESM.bmp]
