# Supplementary material for: Integrative genomics approaches validate PpYUC11-like as candidate gene for the stony hard trait in peach (P. persica L. Batsch)
Source: BMC Plant Biol. 2018 May 18;18:88. doi: 10.1186/s12870-018-1293-6 (PMC5960097; doi:10.1186/s12870-018-1293-6)
Supplement: Supplementary file 3 — Table S2. List of primers used for quantitative PCR analyses. (DOCX 12 kb) [file 12870_2018_1293_MOESM3_ESM.docx]

| **Transcript** | **Simillarity** | **Primer Forward 5'-3'** | **Primer Reverse 5'-3'** |
| --- | --- | --- | --- |
| Prupe.6G163400 | Actin-7 family | GATTCCGGTGCCCAGAAGT | CCAGCAGCTTCCATTCCAA |
| Prupe.6G157400 | YUC11-like | GGACTTCCAAGACAAAGTTTCC | TTGACCGTTGACCTTCTTCAGT |
| Prupe.6G157500 |  |  |  |
| Prupe.6G159800 | RNA-helicase (DHX16) | ATAGCTGAAACTTCGTTGACCATT | AGATGGGAGTAACTAGCAATGACT |
| Prupe.6G160300 | Nodulin-Mt21 | TCTTGCCATGATCTTTGAAAGGAA | TGTAGGTAAGGGCAATGCAATAAG |
| Prupe.6G163500 | MSH3-like | ATCTTCCAAACCATCCAAACTACC | AGAAGGAGAAGGTTTTGGAATGTC |
