# Supplementary material for: Integrative genomics approaches validate PpYUC11-like as candidate gene for the stony hard trait in peach (P. persica L. Batsch)
Source: BMC Plant Biol. 2018 May 18;18:88. doi: 10.1186/s12870-018-1293-6 (PMC5960097; doi:10.1186/s12870-018-1293-6)
Supplement: Supplementary file 4 — Table S3. SRA accession number of assembled Illumina Whole-Genome libraries. (DOCX 13 kb) [file 12870_2018_1293_MOESM4_ESM.docx]

| **accession** | **SRR** | **Systematic Name** |
| --- | --- | --- |
| D41-62 | in submission | *P. persica* (L.) Batsch |
| Jing Yu | SRR830547 | *P. persica* (L.) Batsch |
| Yumyeong | SRR502994 | *P. persica* (L.) Batsch |
| Earligold | SRR502995 | *P. persica* (L.) Batsch |
|  | SRR502996 |  |
| Oro A | SRR502986 | *P. persica* (L.) Batsch |
| Bolero | SRR501836 | *P. persica* (L.) Batsch |
| Elberta | SRR1994378 | *P. persica* (L.) Batsch |
| F1 Contender x Ambra | SRR502997 | *P. persica* (L.) Batsch |
| Georgia Belle | SRR068359 | *P. persica* (L.) Batsch |
| Harrow Blood | SRR1994351 | *P. persica* (L.) Batsch |
| Mayfire | SRR830548 | *P. persica* (L.) Batsch |
| Okitsu | SRR830521 | *P. persica* (L.) Batsch |
| Okubo | SRR830524 | *P. persica* (L.) Batsch |
| Quetta | SRR502987 | *P. persica* (L.) Batsch |
|  | SRR502989 |  |
| Redhaven | SRR1994379 | *P. persica* (L.) Batsch |
| Shu Guang | SRR3237761 | *P. persica* (L.) Batsch |
| Venus | SRR1867740 | *P. persica* (L.) Batsch |
|  | SRR1867794 |  |
|  | SRR1867795 |  |
| Yang Zhou 3 | SRR1994371 | *P. persica* (L.) Batsch |
| Zhong You Tao 4 | SRR1994354 | *P. persica* (L.) Batsch |
