# Supplementary material for: Integrative genomics approaches validate PpYUC11-like as candidate gene for the stony hard trait in peach (P. persica L. Batsch)
Source: BMC Plant Biol. 2018 May 18;18:88. doi: 10.1186/s12870-018-1293-6 (PMC5960097; doi:10.1186/s12870-018-1293-6)
Supplement: Supplementary file 7 — Table S4. List of candidate genes identified on the chromosome 6 region comprised between SNP_IGA_652659 (13,743,178 bp) and SNP_IGA_534275 (15,609,595 bp). (DOCX 17 kb) [file 12870_2018_1293_MOESM7_ESM.docx]

| **Transcript name** | **Arabidopsis homology** | **E-value** |
| --- | --- | --- |
| Prupe.6G156700.1 | APK2B \| protein kinase 2B | 8,20E-126 |
| Prupe.6G156800.1 | FUS12, COP12, CSN2 \| proteasome family protein | 0 |
| Prupe.6G156900.1 | CIPK3 \| CBL-interacting protein kinase 3 | 0 |
| Prupe.6G157300.1 | DNA gyrase B and HSP90-like ATPase family protein | 0 |
| Prupe.6G157400.1 | YUC10 \| Flavin-containing monooxygenase family protein | 2,91E-41 |
| Prupe.6G157500.1 | YUC10 \| Flavin-containing monooxygenase family protein | 2,90E-52 |
| Prupe.6G157800.1 | NDH-O \| NAD(P)H:plastoquinone dehydrogenase complex subunit O | 2,96E-47 |
| Prupe.6G157900.1 | Amino acid kinase family protein | 8,99E-128 |
| Prupe.6G158000.1 | Ribosomal protein L17 family protein | 2,72E-65 |
| Prupe.6G158300.1 | S-locus lectin protein kinase family protein | 1,04E-138 |
| Prupe.6G158400.1 | S-locus lectin protein kinase family protein | 1,32E-121 |
| Prupe.6G158500.1 | Ribonuclease H-like superfamily protein | 6,67E-40 |
| Prupe.6G158700.1 | ARK1 \| receptor kinase 1 | 9,57E-116 |
| Prupe.6G158800.1 | S-locus lectin protein kinase family protein | 2,11E-85 |
| Prupe.6G158900.1 | RLK1 \| receptor-like protein kinase 1 | 3,92E-32 |
| Prupe.6G159000.1 | S-locus lectin protein kinase family protein | 2,85E-138 |
| Prupe.6G159100.1 | Zinc finger C-x8-C-x5-C-x3-H type family protein | 7,51E-25 |
| Prupe.6G159200.1 | IND1 \| bHLH DNA-binding superfamily protein | 6,54E-36 |
| Prupe.6G159400.1 | FAS2 \| Transducin/WD40 repeat-like superfamily protein | 0 |
| Prupe.6G159500.1 | Transducin/WD40 repeat-like superfamily protein | 1,93E-108 |
| Prupe.6G159600.1 | CMPG2 \| CYS, MET, PRO, and GLY protein 2 | 2,27E-118 |
| Prupe.6G159700.1 | IDL4 \| inflorescence deficient in abscission (IDA)-like 4 | 7,79E-11 |
| Prupe.6G159800.1 | ESP3 \| RNA helicase family protein | 0 |
| Prupe.6G159900.1 | RNR1 \| ribonucleotide reductase 1 | 0 |
| Prupe.6G160000.1 | LCV2 \| like COV 2 | 5,90E-93 |
| Prupe.6G160100.1 | disease resistance protein (TIR-NBS-LRR class), putative | 1,17E-162 |
| Prupe.6G160300.1 | nodulin MtN21 /EamA-like transporter family protein | 5,51E-89 |
| Prupe.6G160500.1 | EFR \| EF-TU receptor | 2,83E-42 |
| Prupe.6G160600.1 | Alpha/beta hydrolase related protein | 6,69E-11 |
| Prupe.6G160700.1 | MDAR1 \| monodehydroascorbate reductase 1 | 1,11E-34 |
| Prupe.6G160800.1 | Leucine-rich repeat protein kinase family protein | 1,94E-140 |
| Prupe.6G160900.1 | Leucine-rich repeat protein kinase family protein | 2,31E-126 |
| Prupe.6G161000.1 | Splicing factor, CC1-like | 2,28E-158 |
| Prupe.6G161100.1 | Alfa DNA/RNA-binding protein | 2,79E-53 |
| Prupe.6G161300.1 | CESA6 \| cellulose synthase 6 | 0 |
| Prupe.6G161400.1 | catalytic LigB subunit of aromatic ring-opening dioxygenase family | 4,44E-98 |
| Prupe.6G161500.1 | Transducin/WD40 repeat-like superfamily protein | 1,79E-147 |
| Prupe.6G161700.1 | Protein kinase family protein with leucine-rich repeat domain | 0 |
| Prupe.6G161800.1 | CAD9 \| cinnamyl alcohol dehydrogenase 9 | 1,74E-55 |
| Prupe.6G162000.1 | CAD9 \| cinnamyl alcohol dehydrogenase 9 | 9,90E-153 |
| Prupe.6G162100.1 | FAD/NAD(P)-binding oxidoreductase family protein | 1,54E-85 |
| Prupe.6G162200.1 | microfibrillar-associated protein-related | 1,79E-107 |
| Prupe.6G162400.1 | Chaperone DnaJ-domain superfamily protein | 3,43E-30 |
| Prupe.6G162600.1 | GT2 \| Duplicated homeodomain-like superfamily protein | 4,88E-51 |
| Prupe.6G162800.1 | Cyclin family protein | 3,25E-23 |
| Prupe.6G162900.1 | BolA-like family protein | 1,71E-34 |
| Prupe.6G163000.1 | CLV1 \| Leucine-rich receptor-like protein kinase family protein | 0 |
| Prupe.6G163100.1 | Plastid-lipid associated protein PAP / fibrillin family protein | 3,69E-76 |
| Prupe.6G163200.1 | Ribosomal protein L18e/L15 superfamily protein | 3,27E-96 |
| Prupe.6G163300.1 | CARA \| carbamoyl phosphate synthetase A | 0 |
| Prupe.6G163400.1 | ACT7 \| actin 7 | 0 |
| Prupe.6G163500.1 | MSH3 \| DNA repair protein | 4,25E-42 |
