# Supplementary material for: Integrative genomics approaches validate PpYUC11-like as candidate gene for the stony hard trait in peach (P. persica L. Batsch)
Source: BMC Plant Biol. 2018 May 18;18:88. doi: 10.1186/s12870-018-1293-6 (PMC5960097; doi:10.1186/s12870-018-1293-6)
Supplement: Supplementary file 9 — Table S5. Phenotypic evaluation for SH trait and allelic status at YUC11 TC microsatellites of the two F2 segregating progenies BO10040 and BO10039 (issued from the SH parent ‘D4162’). (DOCX 16 kb) [file 12870_2018_1293_MOESM9_ESM.docx]

| **Seedling** | ***hd* locus** | **Allele size** | **Texture phenotype** |
| --- | --- | --- | --- |
| **Cross Population BO1039** | | | |
| BO1039001 | *Hd/hd* | 104/97 | Melting |
| BO1039002 | *hd/hd* | 97/97 | **Stony hard** |
| BO1039003 | *hd/hd* | 97/97 | **Stony hard** |
| BO1039004 | *Hd/hd* | 104/97 | Melting |
| BO1039005 | *Hd/Hd* | 104/104 | Melting |
| BO1039006 | *Hd/Hd* | 104/104 | Melting |
| BO1039007 | *Hd/hd* | 104/97 | Melting |
| BO1039008 | *Hd/hd* | 104/97 | Melting |
| BO1039009 | *Hd/hd* | 104/97 | Melting |
| BO1039010 | *Hd/hd* | 104/97 | Melting |
| BO1039011 | *Hd/Hd* | 104/104 | Melting |
| BO1039012 | *Hd/hd* | 104/97 | Melting |
| BO1039013 | *Hd/hd* | 104/97 | Melting |
| BO1039014 | *Hd/hd* | 104/97 | Melting |
| BO1039015 | *Hd/hd* | 104/97 | Melting |
| BO1039016 | *hd/hd* | 97/97 | **Stony hard** |
| BO1039017 | *Hd/Hd* | 104/104 | Melting |
| BO1039018 | *hd/hd* | 97/97 | **Stony hard** |
| BO1039019 | *Hd/hd* | 104/97 | Melting |
| BO1039020 | *hd/hd* | 97/97 | **Stony hard** |
| BO1039021 | *Hd/Hd* | 104/104 | Melting |
| BO1039022 | *Hd/hd* | 104/97 | Melting |
| BO1039023 | *Hd/Hd* | 104/104 | Melting |
| BO1039024 | *Hd/Hd* | 104/104 | Melting |
| BO1039025 | *Hd/hd* | 104/97 | Melting |
| BO1039026 | *Hd/hd* | 104/97 | Melting |
| BO1039027 | *Hd/hd* | 104/97 | Melting |
| BO1039028 | *hd/hd* | 97/97 | **Stony hard** |
| BO1039029 | *Hd/Hd* | 104/104 | Melting |
| BO1039030 | *Hd/hd* | 104/97 | Melting |
| BO1039031 | *Hd/Hd* | 104/104 | Melting |
| BO1039032 | *Hd/hd* | 104/97 | Melting |
| BO1039034 | *hd/hd* | 97/97 | **Stony hard** |
| BO1039035 | *hd/hd* | 97/97 | **Stony hard** |
| BO1039036 | *Hd/hd* | 104/97 | Melting |
| BO1039037 | *Hd/Hd* | 104/104 | Melting |
| BO1039038 | *Hd/hd* | 104/97 | Melting |
| BO1039039 | *Hd/hd* | 104/97 | Melting |
| BO1039040 | *Hd/hd* | 104/97 | Melting |
| BO1039041 | *Hd/Hd* | 104/104 | Melting |
| BO1039042 | *Hd/Hd* | 104/104 | Melting |
| BO1039043 | *hd/hd* | 97/97 | **Stony hard** |
| BO1039044 | *Hd/Hd* | 104/104 | Melting |
| BO1039045 | *hd/hd* | 97/97 | **Stony hard** |
| BO1039046 | *Hd/Hd* | 104/104 | Melting |
| BO1039047 | *Hd/Hd* | 104/104 | Melting |
| BO1039048 | *hd/hd* | 97/97 | **Stony hard** |
| **Cross Population BO1004** | | | |
| BO1004001 | *hd/hd* | 97/97 | **Stony hard** |
| BO1004002 | *Hd/hd* | 104/97 | Melting |
| BO1004003 | *Hd/hd* | 104/97 | Melting |
| BO1004004 | *Hd/Hd* | 104/104 | Melting |
| BO1004006 | *Hd/hd* | 104/97 | Melting |
| BO1004009 | *Hd/Hd* | 104/104 | Melting |
| BO1004010 | *Hd/hd* | 104/97 | Melting |
| BO1004015 | *Hd/hd* | 104/97 | Melting |
| BO1004016 | *hd/hd* | 97/97 | **Stony hard** |
| BO1004017 | *Hd/hd* | 104/97 | Melting |
| BO1004018 | *Hd/Hd* | 104/104 | Melting |
| BO1004020 | *hd/hd* | 97/97 | **Stony hard** |
| BO1004022 | *hd/hd* | 97/97 | **Stony hard** |
| BO1004023 | *Hd/Hd* | 104/104 | Melting |
| BO1004024 | *hd/hd* | 97/97 | **Stony hard** |
| BO1004025 | *Hd/hd* | 104/97 | Melting |
| BO1004026 | *hd/hd* | 97/97 | **Stony hard** |
| BO1004028 | *Hd/hd* | 104/97 | Melting |
| BO1004029 | *hd/hd* | 97/97 | **Stony hard** |
| BO1004030 | *Hd/hd* | 104/97 | Melting |
| BO1004031 | *Hd/hd* | 104/97 | Melting |
| BO1004033 | *Hd/Hd* | 104/104 | Melting |
| BO1004034 | *hd/hd* | 97/97 | **Stony hard** |
| BO1004035 | *Hd/Hd* | 104/104 | Melting |
| BO1004036 | *hd/hd* | 97/97 | **Stony hard** |
| BO1004038 | *hd/hd* | 97/97 | **Stony hard** |
| BO1004039 | *Hd/hd* | 104/97 | Melting |
| BO1004040 | *hd/hd* | 97/97 | **Stony hard** |
| BO1004041 | *Hd/hd* | 104/97 | Melting |
| BO1004042 | *Hd/Hd* | 104/104 | Melting |
| BO1004043 | *Hd/hd* | 104/97 | Melting |
| BO1004044 | *Hd/hd* | 104/97 | Melting |
| BO1004045 | *Hd/Hd* | 104/104 | Melting |
| BO1004046 | *Hd/Hd* | 104/104 | Melting |
| BO1004047 | *hd/hd* | 97/97 | **Stony hard** |
| BO1004048 | *hd/hd* | 97/97 | **Stony hard** |
| BO1004049 | *Hd/hd* | 104/97 | Melting |
| BO1004051 | *Hd/hd* | 104/97 | Melting |
| BO1004053 | *Hd/hd* | 104/97 | Melting |
| BO1004054 | *Hd/hd* | 104/97 | Melting |
| BO1004055 | *Hd/hd* | 104/97 | Melting |
